# Enzyme immunoassays for detection and quantification of venoms of Sri Lankan snakes: Application in the clinical setting

**DOI:** 10.1371/journal.pntd.0008668

**Published:** 2020-10-05

**Authors:** Kalana Prasad Maduwage, Indika Bandara Gawarammana, José María Gutiérrez, Chaminda Kottege, Rohana Dayaratne, Nuwan Prasada Premawardena, Sujeewa Jayasingha

**Affiliations:** 1 Department of Biochemistry, Faculty of Medicine, University of Peradeniya, Peradeniya, Sri Lanka; 2 Department of Medicine, Faculty of Medicine, University of Peradeniya, Peradeniya, Sri Lanka; 3 Instituto Clodomiro Picado, Facultad de Microbiología, Universidad de Costa Rica, San José, Costa Rica; 4 District General Hospital, Monaragala, Sri Lanka; Liverpool School of Tropical Medicine, UNITED KINGDOM

## Abstract

**Background:**

Detection and quantification of snake venom in envenomed patients’ blood is important for identifying the species responsible for the bite, determining administration of antivenom, confirming whether sufficient antivenom has been given, detecting recurrence of envenoming, and in forensic investigation. Currently, snake venom detection is not available in clinical practice in Sri Lanka. This study describes the development of enzyme immunoassays (EIA) to differentiate and quantify venoms of Russell’s viper (*Daboia russelii*), saw-scaled viper (*Echis carinatus*), common cobra (*Naja naja*), Indian krait (*Bungarus caeruleus*), and hump-nosed pit viper (*Hypnale hypnale*) in the blood of envenomed patients in Sri Lanka.

**Methodology / Principal findings:**

A double sandwich EIA of high analytical sensitivity was developed using biotin-streptavidin amplification for detection of venom antigens. Detection and quantification of *D*. *russelii*, *N*. *naja*, *B*. *caeruleus*, and *H*. *hypnale* venoms in samples from envenomed patients was achieved with the assay. Minimum (less than 5%) cross reactivity was observed between species, except in the case of closely related species of the same genus (i.e., *Hypnale*). Persistence/ recurrence of venom detection following *D*. *russelii* envenoming is also reported, as well as detection of venom in samples collected after antivenom administration. The lack of specific antivenom for *Hypnale* sp envenoming allowed the detection of venom antigen in circulation up to 24 hours post bite.

**Conclusion:**

The EIA developed provides a highly sensitive assay to detect and quantify five types of Sri Lankan snake venoms, and should be useful for toxinological research, clinical studies, and forensic diagnosis.

## Author summary

Snakebite is a major medical and public health problem in tropical agricultural world. Detection of the type of snake venom and measurement of venom levels in blood are important for snakebite research, selecting the appropriate antivenom, and assessing venom levels in blood at the clinical setting. Currently, a snake venom detection platform is not available in clinical practice in Sri Lanka. This study aimed to develop a double sandwich enzyme immunoassays (EIA) to differentiate and quantify venoms of Russell’s viper (*Daboia russelii*), saw-scaled viper (*Echis carinatus*), common cobra (*Naja naja*), Indian krait (*Bungarus caeruleus*), and hump-nosed pit viper (*Hypnale hypnale*) in blood samples of envenomed patients in Sri Lanka. The EIA developed used biotin-streptavidin amplification for detection of venom antigens and showed high analytical sensitivity. The assay allowed the quantification of venoms of the five species in blood samples from envenomed patients. Low level of cross reactivity was noted between species, except in the case of closely related *Hypnale* species. The presence of *D*. *russelii* venom after antivenom treatment is reported, a finding that has implications in the dosing of antivenom in these envenomings. Lack of specific antivenom for *H*. *hypnale* envenoming offered an opportunity of study the remaining venom antigen in circulation up to 24 hr post bite. The EIA developed constitutes a useful tool to detect and quantify the five types of Sri Lankan snake venoms, and should be useful for research purposes, as well as for the diagnosis and therapy evaluation of clinical cases of envenomings in this country, and for forensic purposes.

## Introduction

Snakebite is a major medical and public health problem in the tropical agricultural world, with estimated annual global burden of 1.8 to 2.7 million and mortality ranging from 81,410 to 137,880 fatalities per year [1, 2]. The World Health Organization (WHO) has classified snakebite as a category A neglected tropical disease, highlighting its global health impact [3].

Snakebite envenoming is more prevalent in south and south-east Asia than in any other region of the world [1, 3]. Sri Lanka is rated amongst countries with the highest snakebite incidence globally. A recent community-based study estimated the annual number of snakebites in Sri Lanka as high as 80,000, with approximately 400 deaths [4]. There are 31,000–40,000 of snake bite victims have been admitted to government hospitals annually in Sri Lanka during the last decade, with approximately 100 deaths [5, 6]. Out of total 106 snake species, life threatening envenoming and fatalities have been reported mostly following bites by Indian krait (*Bungarus caeruleus*), common cobra (*Naja naja*), Russell’s viper (*Daboia russelii*), saw-scaled viper (*Echis carinatus*), and hump-nosed pit vipers (*Hypnale* species) [6, 7]. In addition, Ceylon krait (*Bungarus ceylonicus*), green pit viper (*Trimeresurus trigonocephalus*), and sea snakes cause a low number of clinically relevant bites in the country [6]. Hump-nosed pit vipers (*Hypnale* species) are responsible for the highest number of snakebites in Sri Lanka, with an estimated number of 30,000 cases annually [6].

In Sri Lanka, snakebite envenoming is treated with Indian polyvalent antivenom, which is raised against four highly venomous snake species inhabiting India. Clinical use of Indian polyvalent antivenom in Sri Lanka shows a high incidence of adverse reactions and limited efficacy [8–12]. Indian polyvalent antivenom is not indicated in envenoming by hump-nosed pit viper (*Hypnale* sp), since this venom is not included in the immunizing mixture. Hence, systemic envenoming by hump-nosed pit viper is treated symptomatically.

Snake venom detection in serum samples collected from the snake bite victims has been performed in various parts of the world for diagnostic and research purposes [13–19]. Major clinical problem in managing snakebite envenoming include the lack of accurate identification of the offending snake and the diagnosis of envenoming. Sri Lanka does not have a snake venom detection kit for use in hospitals. A large number of snakebite victims present to health facilities without bringing the offending snake [20]. Even if the snake specimen is available, due to the morphological resemblance, hump-nosed pit vipers are often misidentified as juveniles of Russell’s vipers and saw-scaled vipers [21–23]. Identification of the snake on the basis of descriptions provided by the victims or their companions, or recognition by observation of preserved specimens and pictures, are frequently used but often unreliable methods [20, 24, 25]. Another approach is syndromic identification of the offending snake but the sensitivity and specificity of these methods have limitations [20, 26].

The inability to identify the snake hampers both clinical management and research in toxinology. Incorrect identification of the offending snake leads to inappropriate management of patients, with the consequent under treatment and over treatment associated with adverse outcomes [20, 27]. Moreover, such incorrect identification precludes the characterization of the clinical manifestations of envenomings by different species. Clinical studies, from case reports to randomized controlled trials, are flawed by the inability to confirm the responsible snake species. [28].

Few clinical studies have detected venom of Sri Lankan snakes by Enzyme Immunoassays (EIA), but they have not been introduced in the routine clinical practice [29–33]. The most commonly used method of venom detection in biological samples is EIA owing primarily to its high analytical sensitivity. Since its introduction in 1977 [18], this method has been increasingly used in clinical toxinology research and for routine diagnostic purposes in many countries [14, 34–36].

The aim of this study was to develop a highly sensitive EIA to detect and quantify venoms of Sri Lankan Russell’s viper (*Daboia russelii*), saw scaled viper (*Echis carinatus*), common krait (*Bungarus caeruleus*), common cobra (*Naja naja*), and hump-nosed pit viper (*Hypnale hypnale*) venom in humans, with enough accuracy to distinguish envenoming from background absorbance at picogram venom concentrations. In addition, the study was aimed to evaluate cross-reactivity of the assays with other snake venoms in order to assess its specificity. The method developed was applied to the analysis of serum samples collected from patients suffering snakebite envenoming.

## Methods

### Snake venoms

Venoms from Indian krait (*Bungarus caeruleus*), common cobra (*Naja naja*), Russell’s viper (*Daboia russelii*), saw-scaled viper (*Echis carinatus*), and hump- nosed pit vipers (*Hypnale hypnale*, *H*. *nepa*, *H*. *zara*) were obtained from adult snakes collected in various Sri Lankan localities and kept at the serpentarium of Animal Venom Research International (AVRI), Kandalama, Sri Lanka (with approval from the Department of Wildlife Conservation, approval number: WL/3/2/1/7). Once collected, venoms were freeze-dried and stored at -20°C until further use. For each species, the minimum number of specimens used to prepare the venom pools was 20.

### Immunisation of rabbits and collection of plasma samples

New Zealand white rabbits (3–6 months age and 1.5–2.0 kg body weight) were supplied by the Medical Research Institute, Sri Lanka, and maintained at the animal house, Faculty of Medicine, University of Peradeniya. Rabbits were housed in individual cages, received food and water *ad libitum*, and were kept under a 12:12 hr light: dark cycles. Groups of three rabbits for each venom were initially immunised intramuscularly with venoms of the five species. Immunising mixtures were prepared by mixing and emulsifying 1 ml of a solution of 400 μg venom/ml 0.15 M NaCl (saline solution) and 1 ml complete Freund’s adjuvant (CFA). For each rabbit, 250 μl of the emulsion, containing 50 μg venom, were injected intramuscularly in the thigh. Then, three additional intramuscular venom injections (50 μg per injection) were administered with venom emulsified in incomplete Freund’s adjuvant (IFA) at two-week intervals [37]. The antibody titers were monitored every two weeks by indirect EIA. The rest of booster immunisations were done by injecting intramuscularly 50 μg of venom, in 250 μl of saline solution, every two weeks. After the 6th immunisation, 10 ml of blood samples were collected every two weeks (before booster immunisation) from the marginal ear vein and used for the experiments. Sodium citrate was used as anticoagulant. Blood samples were centrifuged for 10 min and plasma was collected and stored at -45°C until purification of antibodies.

### Purification of antibodies

Purification of antibodies was achieved by affinity chromatography on a protein G Sepharose gel (Abcam catalogue number: ab193260, Cambridge, UK). One ml of immune rabbit plasma was mixed with 1 ml of binding buffer (0.05M sodium acetate, pH 5.0) and filtered through 0.45 μm pore membrane (Acrodisc Syringe Filters, Pall Life Sciences, New York, USA). Diluted rabbit plasma was added to protein G Sepharose under gravitational pressure in binding buffer. The absorbance of flow-through was measured at 280 nm using UV Detector (Model 280, Spectrum Laboratories, California, USA). Bound fraction was eluted with elution buffer (0.1M Glycine, pH 2.5). The eluted fraction was immediately neutralised with 2M Na_2_HPO_4_ buffer (100 μl/ml of fraction). Collected fractions were concentrated 5 times using Vivapore absorbance concentrators (12 kDa MWCO). Concentrated antibody samples were dialysed with Phosphate Buffered Saline [PBS] using a dialysis tubing (Biotech RC, 12 kDa MWCO) overnight at 4–8°C. Antibody fractions were stored at 4°C when used within 2 weeks or at -45°C for long term use.

### Determination of antibody titers by indirect EIA

High binding micro-plate (Greiner Bio-One International, Germany) wells were coated with 100 μl of solutions of each venom (1 μg/ml) in 50 mM Na_2_CO_3_ pH 9.5 buffer. Plates were incubated 1 hr at room temperature and overnight at 4°C. The supernatant was discarded and plates were washed three times with washing buffer (PBS containing 0.5% Tween-20). Then plates were blocked with 1.0% bovine serum albumin (Serox Biosciences, Germany, SRX6154) in PBS, and washed three times with washing solution. One hundred microliters of rabbit plasma samples, diluted 1:500 in 1% BSA in PBS, were added and incubated 1 hr at room temperature. After washing wells three times using washing solution, 100 μl of a 1:4000 dilution of goat anti-rabbit IgG-horseradish peroxidase (HRP) (ab7090) was added. After 1 hr incubation at room temperature, wells were washed three times using washing solution. Then, 100 μl of 3,3’,5,5-tetramethylbenzidine (TMB) was added, and the reaction stopped at 3 min by adding 50 μl 1M H_2_SO_4_. Absorbances at 450 nm were recorded in a microplate reader (Huma Reader HS, Human Diagnostics, Wiesbaden, Germany). All experiments were performed in triplicates.

### Biotinylation of venom specific rabbit polyclonal antibodies

Purified venom specific rabbit polyclonal antibodies were biotinylated using EZ-Link Sulfo-NHS-LC-Biotin (Pierce # 21335). Briefly, 200 μg of polyclonal antibodies in PBS were mixed with 1 ml of 1 mg/ml EZ-Link Sulfo-NHS-LC-Biotin, and kept for 30 min at room temperature. Then the mixture was dialysed in PBS overnight at 4–8°C using 12 kDa MWCO dialysis bags (Spectrum Laboratories). Collected biotinylated antibodies were stored at 4–8°C until used.

### Determination of venom cross-reactivity

Venom cross reactivity of the EIA was assessed by coating micro-plates with rabbit antibodies raised against each particular venom. Serial dilutions (10, 5, 2.5, 1.25, 0.62, 0.31. 0.15 ng/mL) of venoms of the other snake species were prepared in PBS containing 1.0% BSA and added to the plate. After 1 hr incubation at room temperature, secondary biotinylated antibodies were added and incubated for another hour. Streptavidin horseradish peroxidase conjugate and TMB were then used to detect bound venom antigens, and the reaction stopped by adding 50 μl 1M H_2_SO_4_. Absorbances at 450 nm were recorded as described. All experiments were performed in triplicates. Venom concentrations were estimated against absorbance of a standard venom curve of the coated antibody matched venom. Mean venom cross reactivity was calculated and variance was expressed as standard error of the means.

### Venom- antibody binding

Solutions of increasing concentrations of antibodies against each venom (0 to 500 μg/mL) in blocking solution were incubated with 100 ng of corresponding venoms of each snake species for one hour at room temperature. Unbound venom antigens were detected using a sandwich EIA. All samples were measured in triplicate, and the average absorbance at 450 nm was converted to a concentration of the venom of interest by comparison with a standard curve based on serial dilutions of venom. Antibody binding to venom was expressed as median effective concentration (EC_50_), defined as the antibody dilution that decreased measured venom concentration by 50% [12]. Mean venom–antibody bindings are presented and variances are expressed as the standard error of means. S1 File summarizes the procedures followed in the study.

### Collection of clinical data and samples

Clinical data and serum samples were collected from patients admitted to the Toxicology Ward, Teaching Hospital, Peradeniya and District General Hospital, Monaragala, with a history of snakebite. Basic demographic, clinical and antivenom treatment data were recorded into standard data collection sheet. After obtaining signed, informed consent, 5 ml of blood was collected by venipuncture in glass tubes and allowed to clot at room temperature. Then they were centrifuged at 3,000 xg at 4°C for 10 min, and serum samples were collected. Serum samples were sent to the laboratory in the Faculty of Medicine, University of Peradeniya and stored at -45°C until analysis. Culprit snake specimens were identified by the corresponding author (also a herpetologist) using standard identification characters. Furthermore, clinical signs and symptoms of envenoming were correlated with the identified snake species. Samples from 20 patients who were bitten by non-venomous snakes were obtained and used as negative controls.

Local envenoming was diagnosed by the development of severe pain, swelling, blistering and local tissue necrosis. Coagulopathy was diagnosed based on positive 20 min Whole Blood Clotting Test (20WBCT), prolonged prothrombin time (PT) and elevation of the International Normalized Ratio (INR). Renal involvement was diagnosed by the development of oliguria / anuria, haematuria, elevation of serum creatinine and urea concentrations, and changes in serum electrolytes (sodium and potassium) concentrations. Neurotoxicity was diagnosed by the development of ptosis, external ophthalmoplegia, diplopia and respiratory paralysis.

### Venom specific EIA and quantification of snake venom in samples from envenomed patients

Venom specific antibodies (100 μl) were applied to wells at concentration of 1 μl/ml, diluted in 50 mM Na_2_CO_3_ pH 9.5 buffer, and were incubated 1 hr at room temperature, and overnight at 4°C. The supernatant was discarded and plates were washed three times with washing buffer (PBS containing 0.5% Tween-20). Then, plates were blocked with 1.0% BSA in PBS. Wells were washed three times at each step using washing buffer. Standard concentrations of venom or diluted samples of sera from patients were added and incubated 1 hr at room temperature. Bound venom antigens were detected by addition of venom specific biotinylated secondary antibodies, followed by streptavidin horseradish peroxidase conjugate (Streptavidin, HRP conjugate, Merck Millipore) and TMB. The plates were washed three times with washing solution in each step. Finally, the reaction was stopped at 3 min by adding 50 μl 1M H_2_SO_4_. Absorbances at 450 nm were recorded in a microplate reader (Huma Reader HS). The concentration of venom protein in sera was estimated against a standard curve developed using a known concentration series (0–10 ng/ml) of venom [29, 38]. Serum samples from patients were diluted 80 times with 1.0% BSA in PBS solution and 100 μl of diluted samples were incubated 1 hr at room temperature. When no venom was detected using 1: 80 dilution factor, a 1: 10 dilution was used. When absorbance of the patient samples was above the maximum absorbance of the standard curve, venom concentrations were calculated using 1: 400 dilution factors. All experiments were performed in triplicates. The limit of detection (LoD) was also determined according to Kulawickrama et al. (2010) EP17-A protocol [38, 39].

### Detection of venom in serum samples of patients treated with antivenom

Venom antigens in serum samples from patients that had been treated with antivenom were detected after dissociating venom-antivenom complexes with glycine-HCl (pH 2.2) and heating for 30 min at 95°C [31]. Treated serum samples underwent venom EIA as described above [31].

### Ethical statement

Serum samples of envenomed patients were collected at the Teaching Hospital, Peradeniya, Sri Lanka, from February 2017 to February 2018, and the District General Hospital, Monaragala, from October 2019 to March 2020. All study patients are adults and signed an informed consent document approved by the Institutional Ethical Review Committee, Faculty of Medicine, University of Peradeniya (Approval No:2015/EC/87). Experiments involving use of animals were approved by the Institutional Ethical Review Committee, Faculty of Medicine, University of Peradeniya (Approval No: 2016/EC/97). The protocol for rabbit studies was based on guidelines provided by the Council for International Organizations of Medical Sciences (CIOMS) [40].

## Results

### Development of antibody titers in rabbits

**Fig 1:**
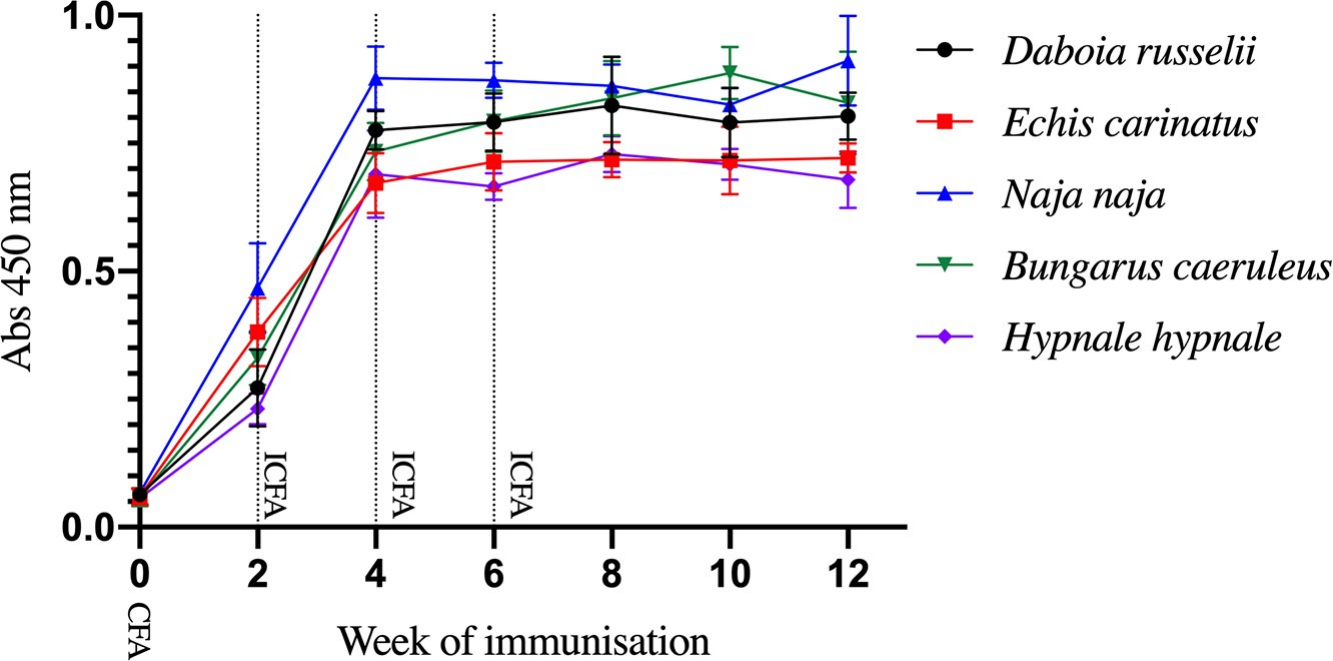
EIA analysis of the time-course of development of antibody response of rabbits immunised with the venoms of Sri Lankan snakes. CFA: Complete Freund Adjuvant; IFA: Incomplete Freund adjuvant.

Venoms from the five species induced a steady and quick rise of antibody titers following the first immunisation with CFA, and after three additional immunisations with IFA. This antibody response indicates that 50 μg of venom is sufficient to generate a high antibody titer in rabbits. Antibody responses to all venoms peaked and then reached a plateau after the third immunization (Fig 1).

### Determination of venom cross reactivity

Cross-reactivities of antibodies raised against each venom, determined by sandwich EIA, are given in Table 1. Antibodies developed against *H*. *hypnale* venom showed high cross reactivity with venoms of other closely related hump-nosed vipers (*H*. *zara* and *H*. *nepa*); 85.3% and 87.4% maximum cross reactivity was observed with venoms of *H*. *zara* and *H*. *nepa*, respectively. Antibodies developed against *D*. *russelii* venom had a maximum of 3.6% and 1.9% cross reactivity with venoms of *N*. *naja* and *B*. *caeruleus*, respectively. There was a higher cross-reactivity with the two other viper venoms (5.3% and 4.9% with *E*. *carinatus* and *H*. *hypnale* venoms, respectively). *B*. *caeruleus* venom antibodies showed the least cross reactivity with other venoms, with a maximum cross reactivity of 5.0% with *N*. *naja* venom. On the other hand, *N*. *naja* venom antibodies showed 4.7% maximum cross-reactivity with *B*. *caeruleus* venom. The venom and antibody cross reactivity matrix demonstrates a low cross-reactivity of less than 5% between all venoms, except with venoms of species within the genus *Hypnale*.

**Table 1:** Percentages of cross reactivity with venoms from several species with the tested venom specific antibody coated double sandwich EIA.

	Cross reactivity percentage									
	Coated and secondary antibody type									
	*Daboia russelii*		*Echis carinatus*		*Naja naja*		*Bungarus caeruleus*		*Hypnale hypnale*	
Tested venom	%	SEM	%	SEM	%	SEM	%	SEM	%	SEM
*Daboia russelii* 10 ng/ml	100		4.8	0.5	1.5	0.3	1.1	0.3	4.1	0.3
*Daboia russelii* 5 ng/ml	100		4.1	1.2	1.7	0.3	1.7	0.7	3.2	0.2
*Echis carinatus* 10 ng/ml	5.3	1.8	100		0.9	0.4	2.6	0.7	4.5	0.4
*Echis carinatus* 5 ng/ml	4.9	1.2	100		1.8	0.7	1.5	0.3	3.6	0.4
*Naja naja* 10 ng/ml	3.6	0.4	2.6	0.1	100		5.0	0.6	2.0	0.2
*Naja naja* 5 ng/ml	2.3	0.4	2.4	0.8	100		4.1	0.6	1.2	0.6
*Bungarus caeruleus* 10 ng/ml	1.0	0.3	1.8	0.3	4.7	0.6	100		1.6	0.3
*Bungarus caeruleus* 5 ng/ml	1.9	0.5	0.9	0.5	4.1	0.6	100		1.5	0.7
*Hypnale hypnale* 10 ng/ml	4.9	0.4	3.7	0.3	2.8	0.5	1.5	0.3	100	
*Hypnale hypnale* 5 ng/ml	3.6	1.0	2.9	0.3	1.3	0.5	2.0	0.4	100	
*Hypnale zara* 10 ng/ml	2.5	0.4	3.0	0.4	1.2	0.3	2.3	0.3	85.3*	6.2
*Hypnale zara* 5 ng/ml	2.2	0.6	3.2	0.5	2.2	0.5	2.7	0.8	76.5*	10.1
*Hypnale nepa* 10 ng/ml	3.5	0.5	2.7	0.3	2.8	0.7	1.4	0.3	87.4*	9.2
*Hypnale nepa* 5 ng/ml	3.8	1.4	3.6	0.6	2.4	0.9	1.8	0.4	72.0*	6.0

SEM: Standard error of mean. The data used in this table is available in (S2 File).

*p < 0.05 when compared with the mean value of anti-*H*. *hypnale* antibodies and *H*. *hypnale* venom. For all other antibodies, a highly significant difference (p < 0.0001) was observed when comparing the mean value obtained with the homologous venom and the values obtained for heterologous venoms.

### Efficacy of antibodies binding to venoms

**Fig 2:**
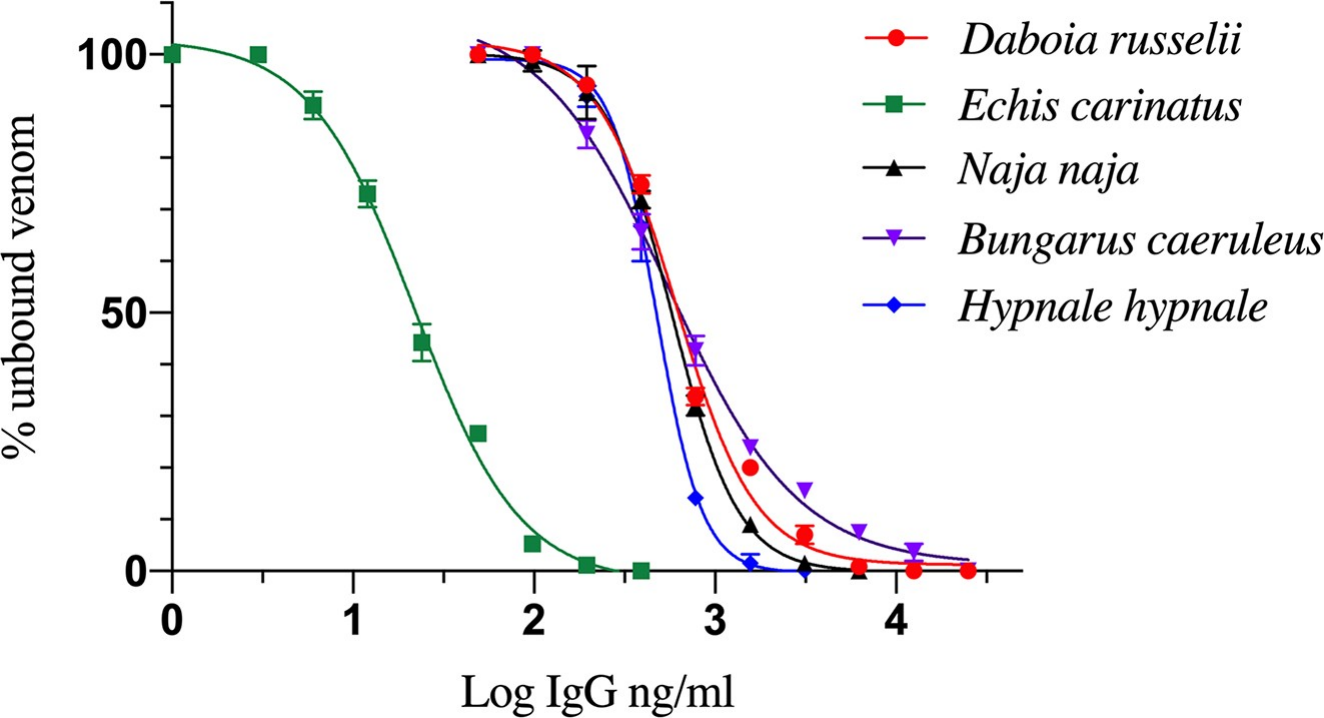
Venom-antibody binding efficacy determined by EIA for the five venoms.

All types of venom specific antibodies effectively bound to their corresponding venom. Percentages of unbound venom while increasing the concentration of antibodies are depicted in Fig 2. Maximum venom binding efficacy was found for antibodies raised against *E*. *carinatus* venom. EC_50_ values of antibodies and venoms of *D*. *russelii*, *E*. *carinatus*, *N*. *naja*, *B*. *caeruleus*, and *H*. *hypnale* were 604.4 ng/ml, 22.0 ng/ml, 575.1 ng/ml, 557.0 ng/ml and 476.8 ng/ml, respectively. Limit of detection (LoD) of venoms of *D*, *russelii*, *E*. *carinatus*, *N*. *naja*, *B*. *caeruleus* and *H*. *hypnale* were 0.87 ng/ml, 1.56 ng/ml, 0.39 ng/ml, 0.19 ng/ml and 1.56 ng/ml, respectively.

### Quantification of snake venom in serum samples from envenomed patients

**Fig 3:**
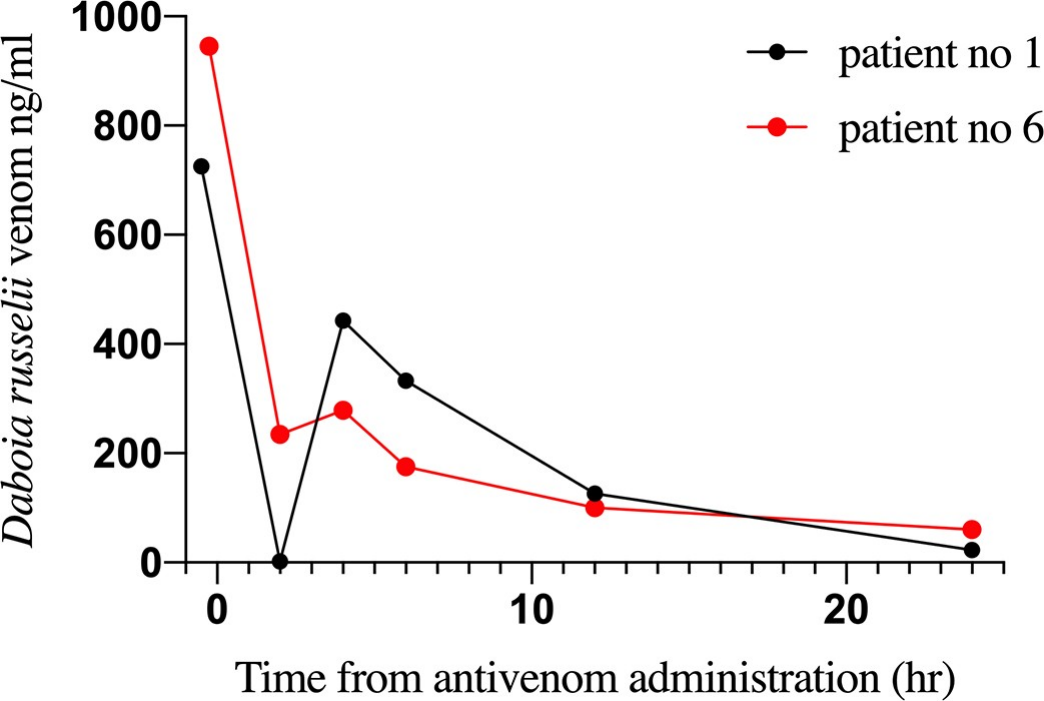
Persistence / recurrence of *D*. *russelii* venom detected by EIA in two patients.

**Fig 4:**
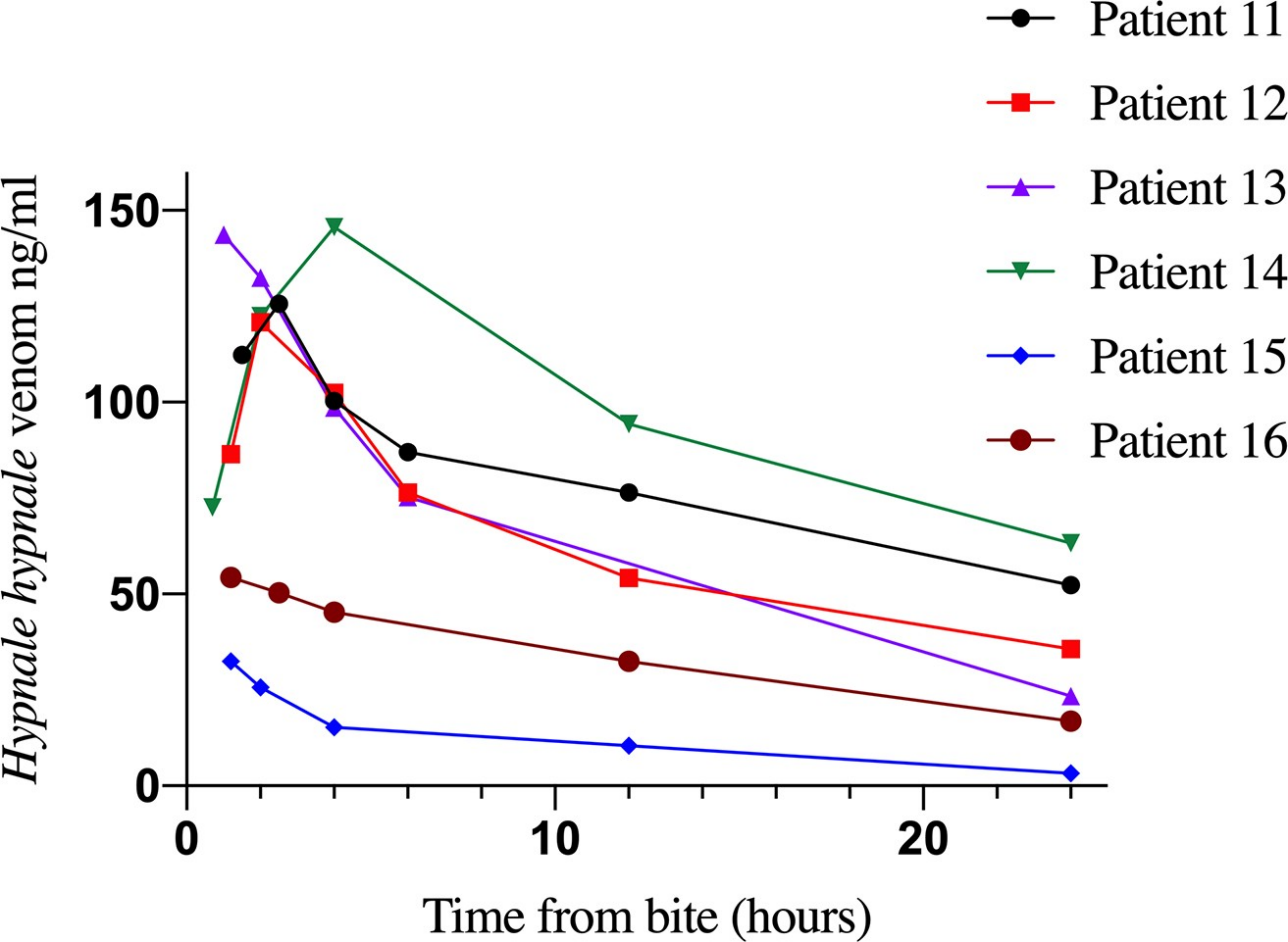
Quantification of *Hypnale* species venom concentrations at different time intervals in six patients.

Clinical data and samples of a total of 39 patients admitted with a history of snakebite were collected and analyzed for the five types of venom EIA. Clinical data, types of detected venom and doses of antivenom administered are depicted in Table 2. Venom was detected by EIA in samples of nineteen patients, and all of them showed clinical manifestations of envenoming. In contrast, none of the patients whose samples rendered negative for venom detection presented clinical evidence of envenoming. *D*. *russelii* venom was detected in 10 patients and all of them developed local and systemic manifestations of envenoming (Table 2). All six patients who were positive for *Hypnale* venom developed local effects and coagulopathy diagnosed based on positive 20WBCT and prolonged prothrombin time tests (Table 2). Detection of *Hypnale* venom in patient samples by EIA was considered as *Hypnale* species venom due to high percentages of cross-reactivity between three species of *Hypnale*, which does not allow the identification of the species. *Hypnale* species, *N*. *naja* and *B*. *caeruleus* venoms were detected in six, two and one patients, respectively. The highest venom concentrations corresponded to patients bitten by *D*. *russelii*, ranging from 31.4 to 945.7 ng/ml. *Hypnale* species venom levels ranged from 32.5 to 143.6 ng/ml. Except for two patients (No. 1 and 6), snake venom was not detected in samples from patients envenomed by *D*. *russelii*, *N*. *naja* and *B*. *caeruleus* and treated with antivenom. In the cases of patients 1 and 6 who had *D*. *russelii* envenoming, venom concentration in serum varied even after antivenom infusion, and venom was detected after 24 hr (Fig 3). In cases of *Hypnale* species bites, in which antivenom was not used, venom was detected in multiple samples collected up to 24 hr from the time of the bite (Fig 4).

**Table 2:** Detection and quantification of snake venom in serum samples collected from patients before antivenom administration. RVV: Russell’s viper (*D*. *russelii*) venom, SSVV: Saw scaled viper (*E*. *carinatus*) venom, CV: Cobra (*N*. *naja*) venom, IKV: Common krait (*B*. *caeruleus*) venom, and HNVV: Hump-nosed pit viper (*Hypnale* species) venom.

Patient No	Age (sex)	Type of envenoming	Time of sample (time from bite, hours)	Type of venom detected	Calculated concentration of venom (ng/ml)	Vials of antivenom given
1	46 (male)	Local, coagulopathy, renal involvement, neurotoxicity	2.5	RVV	725.5	10
2	38 (female)	Local, coagulopathy, neurotoxicity	0.7	RVV	315.4	10
3	49 (male)	Local, coagulopathy, renal involvement, neurotoxicity	1.5	RVV	246.7	10
4	43 (male)	Local, coagulopathy, renal involvement, neurotoxicity	0.8	RVV	187.3	10
5	46 (male)	Local, coagulopathy, neurotoxicity	1.7	RVV	546.0	10
6	20 (male)	Local, coagulopathy	2.5	RVV	945.7	10
7	16 (male)	Local, coagulopathy	1.3	RVV	648.4	10
8	16 (male)	Local, coagulopathy	0.7	RVV	654.9	10
9	44 (male)	Local, coagulopathy	1.8	RVV	325.2	10
10	16 (male)	Local, coagulopathy	2.3	RVV	843.4	10
11	44 (female)	Local, coagulopathy	1.5	HNVV	112.3	0
12	40 (male)	Local, coagulopathy	0.7	HNVV	86.4	0
13	62 (male)	Local, coagulopathy	0.8	HNVV	143.6	0
14	59 (male)	Local, coagulopathy	1.2	HNVV	72.6	0
15	21 (male)	Local, coagulopathy	0.7	HNVV	32.5	0
16	45 (male)	Local, coagulopathy	2.2	HNVV	54.3	0
17	25 (male)	Local, neurotoxicity	2.3	CV	387.5	10
18	62 (male)	Local, neurotoxicity	1	CV	298.5	0
19	56 (female)	Neurotoxicity	0.7	IKV	22.6	10

Samples from patients who were bitten by non-front fanged colubroid snakes, i.e., ‘colubrids’, showed negative results when tested for the presence of the five types of venom and did not show clinical manifestations of envenoming. No samples were available from patients bitten by *E*. *carinatus*, as these cases are restricted to a limited geographical region of the country.

### Detection of venom antigens in post-antivenom samples

A modification of our EIA was introduced in order to detect the presence of venom in samples from patients who had been treated with antivenom. This is based on a combination of acid treatment and heating of serum, which dissociates antigen-antibody complexes, hence allowing qualitative venom detection. This assay was used in the case of eight patients for whom no pre-antivenom samples were available. Samples of five patients were positive for *D*. *russelii* venom and the other three were positive for *Hypnale* species venom.

## Discussion

This study describes a highly sensitive EIA for detection and quantification of the venoms of the five most medically important snakes of Sri Lanka. Compared to previously published studies, the assays developed had a lower LoD, hence having an improved analytical sensitivity. The use of a simple, inexpensive biotinylation step contributed to the increased sensitivity. Minimum (less than 5%) of cross reactivity between venoms was observed, except in the case of closely related species of *Hypnale*. Nevertheless, in these cases the test allows for the identification of the offending genus of snake. There is no clinical relevance of differentiating between *Hypnale* species as they induce identical clinical features [41, 42]. Therefore when antivenom becomes available for *Hypnale*, this test will still be useful in clinical decision making.

EIA based snake venom antigen detection was first described 1977 [18] and has been used in many clinical and laboratory studies. We have increased the sensitivity of the assay by the use of biotin-streptavidin conjugate, which has been previously used for venom assays [38, 43, 44]. The low LoD values described in the current study is similar to a highly sensitive venom assay for taipan (*Oxyuranus* species) venom in Australia [38].

Thus far, only *D*. *russelii*, *B*. *caeruleus* and *H*. *hypnale* venoms have been quantified in serum samples from patients [29, 32, 33, 36, 45]. However, in some of these studies the details of the design of the assays were not described. Our work describes, for the first time, the detection of venoms of Sri Lankan *E*. *carinatus* and *N*. *naja*. A detailed study of development of EIA for *H*. *hypnale* venom was done by Tan et al. (2012), describing the cross reactivity, LoD, and potential clinical applications [37]. In our study we were able to achieve lower LoD for the venoms tested. The high analytical sensitivity of the EIA herein described allows the detection in clinical serum samples of venoms of *E*. *carinatus*, *H*. *hypnale* and *B*. *caeruleus*, which inject relatively small volumes of venom, hence enabling the immunological diagnosis of envenoming by these species.

Cross reactivity of venom and antibodies between closely related species is a common limitation of EIA. However, low percentages (less than 5%) of cross-reactivity were observed in this study. The cross-reactivity between three viperid and two elapid species is not too high and can be easily distinguishable by EIA. Closely similar venom profiles, *in-vivo* toxic effects and clinical manifestations of envenoming have been reported in the cases of the three Sri Lankan species of *Hypnale* [41, 46, 47]. Accordingly, a high extent of cross reactivity was observed between the *H*. *hypnale* antibodies and the venoms of the three species of *Hypnale*. This should not have relevant implications for the application of our EIA in the clinical setting since (a) over 95% of *Hypnale* cases in Sri Lanka are inflicted by *H*. *hypnale* [21, 41, 45], (b) their clinical effects are identical, and (c) the therapeutic approach is the same in cases by the three *Hypnale* species.

Identification of the type of snake venom in patients’ blood is important for epidemiological purposes. Only about 25% of snakebite victims admitted to hospitals bring the culprit specimens, and quite often the identification of the snake is made by showing photographs or by a syndromic approach [20]. None of these approaches are accurate enough, hence limiting the validity of epidemiological studies. Guidelines for the management of snakebite envenoming do not recommend capturing the offending snake and bringing it to the hospital, owing to the risks of bites [48]. Lack of venom confirmation has limited the identification of snakebite cases in many clinical studies [49, 50]. Use of venom EIA would facilitate enrolling all envenomed patients in future clinical studies, hence strengthening the association between snake identification and clinical and therapeutic findings.

Detection and quantification of snake venom in serum is crucial for antivenom dose finding studies. Currently, the selection of antivenom doses in the management of snakebite envenoming in Sri Lanka is based on consensus through clinical experience, as specified in national guidelines [48]. Lack of antivenom dose finding studies is a main reason for inaccurate administration of antivenom. The establishment of appropriate doses of antivenom for various snake species should be based on venom quantification in a cohort of envenomed patients, vis-à-vis the observations on resolution of clinical manifestations of envenoming. Issues such as initial dose, repeated dose, and lapse between two doses of antivenom should be based on the amount of venom present and the relationship between venom toxicokinetics and antivenom pharmacokinetics, together with the clinical evolution of the cases [51]. This type of analysis is largely missing in Sri Lanka. Therefore, quantification of snake venom in the sera of patients is required to establish evidence-based protocols of antivenom administration.

In Sri Lanka, the majority of snakebites are caused by *H*. *hypnale*, and most severe cases and fatalities are due to *D*. *russelii* envenoming [6]. Owing to the fact that envenomings by these species have common initial clinical manifestations [21, 42, 50, 52], identification of the snake responsible is a challenge in clinical practice. Currently administration of Indian polyvalent antivenom is indicated for envenomings by *D*. *russelii*, but not for those inflicted by *Hypnale* sp [48]. The lack of an accurate method for discriminating between these two types of envenoming has led to both under treatment in cases of true *D*. *russelii* bites and unnecessary administration of anitvenom in cases of *Hypnale* sp. This is relevant since Indian antivenom administration often leads to life threatening adverse reactions [9, 11, 53].

*B*. *caeruleus* bites are usually painless and frequently occur at night, people often being unaware of the bite [36, 54]. These envenomings are frequently associated with vague clinical symptoms such as abdominal pain and subtle sings of neurotoxicity which makes clinical diagnosis difficult [36, 54]. On the other extreme, some patients present with complete respiratory paralysis and coma where snake envenoming is listed in the differential diagnosis. Early identification of snake envenoming is important for clinical management and only the detection of venom in patient serum can confirm this diagnosis in these cases.

The implementation of this assay can be used to detect venom in clinical samples in cases of doubt in diagnosis. For instance, the development of complex clinical manifestations following Russell’s viper envenoming is common in Sri Lanka [30, 52]. Delayed admissions due to seeking traditional therapies and other reasons, may result in multi-organ failure following snakebite envenoming, and sometimes the victim has no evidence of a snakebite other than the presence of fang marks. Clinicians usually treat these patients by the suspicion of snakebite envenoming, and often decide to administer antivenom without robust evidence of envenoming. Moreover, a significant number of patients, particularly children, arrive unconscious to hospitals in Sri Lanka [55], as described following *D*. *russelii* and *H*. *hypnale* envenomings [55, 56]. In these situations, the detection of venom in serum samples by EIA should greatly contribute to the appropriate diagnosis and therapy. Moreover, early detection of venom in blood before the onset of clinical features of envenoming is crucial to start an early antivenom treatment and prevent further venom toxicity [57].

Detection of venom in samples collected after antivenom infusion is important in situations where no samples were available prior to antivenom administration. Sometimes, envenomed patients receive the first dose of antivenom before they reach the major hospitals. Hence, snake venom detection is not possible in these cases due to complete neutralization of venom by antivenom. Maduwage et al. (2016) described a method to dissociate venom-antivenom complexes, thus allowing venom detection [31]. This protocol detected *D*. *russelii* and *H*. *hyupnale* venom in post-antivenom samples in our study, indicating a further use of EIA in clinical practice. Even though accurate quantification of venom was not feasible in these circumstances (31), the qualitative detection of venom provides valuable information for diagnosis and management.

Persistence/ recurrence of venom antigen following *D*. *russelii* envenoming has been reported [53, 58, 59], and explained by Maduwage et al. [29] who detected both free and antivenom-bound venom by EIA [60]. The detection of *D*. *russelii* venom antigens in serum samples in two patients after administration of antivenom revealed that they had high concentration of venom in their blood on admission and that the antivenom dosage was insufficient, in agreement with the results of Maduwage et al.[29].

In the case of patients envenomed by *H*. *hypnale*, where no antivenom is currently administered, it is possible to study the toxicokinetics of venom. In our study, venom was detected even 24 hr after the bite. Snake venom toxicokinetics has been described based on two [61] or three compartment models [62]. It is relevant to study *H*. *hypnale* venom toxicokinetics in a larger number of patients in Sri Lanka. Another possible application of EIA in snakebite research has to do with forensic toxicology, in the analysis of cases of sudden deaths of uncertain causality where snakebite is a possibility. EIA of high analytical sensitivity should allow the identification of traces of venom in body fluids or tissue extracts.

A limitation of this study is that the described method of snake venom antigen detection should be conducted in a laboratory by a trained technician and is time consuming. Hence, the described test does not allow the rapid detection of venom at the clinical setting. The development of a rapid venom detection kit, such as an immunochromatography (lateral flow) test, would be a practical solution to detect the venom at the point of care. Our findings provide a proof of concept that it is feasible to specifically detect and quantify venom from the most important snakes in Sri Lanka. Our results offer valuable information for the future development of a rapid point of care venom detection kit in Sri Lanka.

Another limitation is that serum samples from patients bitten by *E*. *carinatus* have not been tested in this study. The limited distribution of *E*. *carinatus* in Sri Lanka leads to lack of access to hospitals where patients are admitted following *E*. *carinatus* bites. Thus, the detection of *E*. *carinatus* venom in clinical samples has to be studied in the future. Moreover, the numbers of patients with *N*. *naja* and *B*. *caeruleus* envenomings were limited in the study because these envenomings are not frequent in Sri Lanka. Analysis of samples from a large cohort of envenomed patients following *E*. *carinatus*, *N*. *naja* and *B*. *caeruleus* envenoming is recommended for assessing the performance of this diagnostic test in clinical cases.

In conclusion, our study constitutes a proof of concept that EIA is a reliable tool to identify the species of Sri Lankan snakes responsible for the bite and to quantify venom concentration in serum before and after antivenom administration. Such methodology could be useful not only in the diagnosis of the offending snake species, but also in the quantification of circulating venom and the assessment of whether the volume of antivenom administered was enough to eliminate venom in blood. Owing to its analytical sensitivity and specificity, it discriminates between venoms from these five species and may become a valuable tool for the diagnostics and clinical management of snakebite envenoming in this country. On the basis of our findings, it should be feasible to design kits based on liquid or lateral flow assays for venom detection that could provide rapid results.

## Supporting information

S1 File Flow chart of the process of the development of enzyme immunoassay for detection and quantification of venoms of Sri Lankan snakes. (DOCX)

S2 File Data use in relation to figure number 1–4 and table number 1 and 2. (XLSX)
