# Supplementary material for: Enzyme immunoassays for detection and quantification of venoms of Sri Lankan snakes: Application in the clinical setting
Source: PLoS Negl Trop Dis. 2020 Oct 5;14(10):e0008668. doi: 10.1371/journal.pntd.0008668 (PMC7561112; doi:10.1371/journal.pntd.0008668)
Supplement: S1 File — (DOCX) [file pntd.0008668.s001.docx]

**Supplementary Figure S1**

**Flow chart of the process of the development of enzyme immunoassay for detection and quantification of venoms of Sri Lankan snakes**

Development of venom specific enzyme immunoassay

Quantification of venom in clinical samples

Determination of venom – antibody binding activity

Determination of cross reactivity of venoms

Biotinylation of species specific polyclonal rabbit antibodies

Purification of antibodies using Protein-G Sepharose affinity chromatography

Determination of antibody titers by indirect EIA

Collection of blood samples after 6 weeks of immunization and separation of serum

Immunization of rabbits with venoms of *D.russelii, E. carinatus, N. naja, B. caeruleus* and *H. hypnale*
